# Kinetic Diffusion Couple for Mapping Microstructural and Mechanical Data on Ti–Al–Mo Titanium Alloys

**DOI:** 10.3390/ma11071112

**Published:** 2018-06-29

**Authors:** Yi Chen, Hongchao Kou, Liang Cheng, Yunlong Zhang, Yun Yu, Yalin Lu

**Affiliations:** 1 School of Materials and Engineering, Jiangsu University of Technology, Changzhou 213001, China; chengliang525@163.com (L.C.); liuding31@163.com (Y.Z.); yuyun@jsut.edu.cn (Y.Y.); jxlyl@jsut.edu.cn (Y.L.); 2 IMDEA Materials Institute, 28040 Madrid, Spain; 3 E.T.S. de Ingenieros de Caminos, Polytechnic University of Madrid, 28040 Madrid, Spain; 4 Sunnywell (China) New Material Technology Co., Ltd., Changzhou 213000, China; 5 State Key Laboratory of Solidification Processing, Northwestern Polytechnical University, Xi’an 710072, China; hchkou@nwpu.edu.cn

**Keywords:** kinetic diffusion couple, Ti-Al-Mo, combinatorial alloy design, microstructural and mechanical characteristics, composition–microstructure–property relationship

## Abstract

We introduce a new strategy that extends the established diffusion couple approach for efficient mapping of microstructural and mechanical properties in bulk samples. The featured diffusion couples undergo an interdiffusion annealing followed by a thermal/mechanical treatment for creating a blended spectrum of phases and microstructures in the well-grooved continuous composition gradients, which is then further accessible to local high spatially resolving microanalysis probing. The strategy is demonstrated on two diffusion couples, Ti/Ti-7.58Al-4.97Mo and Ti-5.04Al/Ti-1.52Mo, by surveying the spectrums of microstructural and mechanical characteristics created through an appreciation of the β-to-α phase transformation with such microanalysis techniques as electron probe microanalysis, electron backscatter diffraction analysis, and nanoindentation. The examined microstructural characteristics and mechanical properties reveal the variation of phase, morphology, and microhardness based on the changes of the compositions of the overall alloys and the individual phases in these near-β Ti alloys.

## 1. Introduction

The requirement of the high-performance airplane accelerates the application of titanium alloys due to their high specific strength together with their extraordinary corrosion resistance [1,2]. For the novel lightweight near-β titanium alloys, the β-to-α diffusive phase transformation governs much of the microstructures and properties [3,4]. Due to the inherent complexity of hexagonal metals and the multicomponent nature of engineering materials, the alloying effects on phase transformations, microstructure, and properties are quite often sensitive, which will increase the difficulty for designing new types of near-β titanium alloys. Thus, the alloy designers pay much attention to the acceleration mapping of the relationship among composition, microstructure, and properties of alloys. Many high throughput methods then have been developed [5,6,7], for example, H. Springer and D. Raabe [6] proposed a new approach named rapid alloy prototyping for the compositional and thermo-mechanical design and rapid maturation of bulk structural materials. 

Nowadays, the diffusion couple technique, a traditional and effective method for rapid screening of phase diagram [8] and diffusion data [9], has been developed as a high throughput method for screening many important materials data [10,11], such as the composition property [12].

In this paper, we make an effort to extend the traditional diffusion couple to the “kinetic diffusion couple” (KDC) technique that can include much microstructure information by combining with thermal/mechanical treatment, which makes it possible to rapidly map thousands of alloys’ microstructures and properties by just one diffusion couple.

The Ti–Al–Mo ternary, the archetypal base system of many important β-Ti alloys like Ti-5553 [13] and β-21S [14], is performed in this study, which gives the example for accelerated alloy design.

## 2. Materials and Methods 

### 2.1. Methodology

In this paper, we introduce a new high throughput method named “kinetic diffusion couple” (KDC) for accelerating discovery and development of the novel titanium alloys [15,16]. As we know, the properties of titanium are determined by the composition and microstructure of the alloys, especially controlled by the β-to-α diffusive phase transformation. The characterization of the alpha phase, including the strength, morphology, and distribution of alpha plays a key role in the final properties of the alloy. Thus, in order to accelerate designing the novel titanium alloys, the final properties of the alloys, including the information about composition, the β-to-α phase transformation process, the microstructure, and the property characteristics of the alpha phase, should be obtained simultaneously. Thanks to the traditional diffusion couple technique, we can get the compositional gradients by long-term annealing. Then, combined with nanoindentation tester, the composition–properties relationship can be obtained rapidly, which has been applied in J.C. Zhao’s [17,18] work. However, the composition–properties relationship is too far away from designing the titanium alloys. Thus, we consider a way to include the microstructure information into this traditional technique. As for the titanium alloy, the heat treatment process will introduce the β-to-α phase transformation process [19]. As a diffusion-controlled phase transformation, the growth of the α phase can be controlling by adjusting the heat treatment process [20], which finally will introduce the microstructure gradients based on certain composition gradients. Then, by combining with the high-resolution experiments, such as the electron microprobe analysis (EPMA), the electron backscatter diffraction (EBSD), nanoindentation, and high resolution scanning electron microscope (HR-SEM), the nanoscale composition, microstructure and property characteristics can be measured. Due to the simultaneous diffusion and kinetic phase transformation process, we define this method as the “kinetic diffusion couple” (KDC) method. The advantages of KDC are notably that many critical materials data, including the precipitation kinetics, solution-strengthening effects, and precipitation-strengthening effects, can be rapidly screened, which is also suitable for the ICME and materials genome.

### 2.2. Diffusion Couple Experiments

Two diffusion couples, Ti/Ti-7.58Al-4.97Mo (at %) and Ti-5.04Al/Ti-1.52Mo (at %), were prepared. Each Ti-based alloy was designed with its nominal composition placed in the β solid solution area of Ti–Al–Mo at 1250 °C according to the accepted phase diagrams [21,22,23], and was prepared from 99.9% sponge Ti, 99.99% Al, and 99.9% Mo (mass%) by arc melting in an argon atmosphere. The arc melting was repeated ten times to attain a homogeneous composition. The ingots were solid-solutioned at 1200 °C for 8 h under vacuum followed by water-quenching. The solution treatment resulted in the alloys with average grain size larger than several millimeters such that the effect of grain boundary diffusion could be ignored. Small cylinder samples for diffusion couples were cut from the ingots into a size of φ15 mm × 5 mm by using wire-electrode cutting. Small disks were prepared by polishing one surface of the cylinder samples and annealed pure Ti to mirror-like quality. The well-contacted diffusion couples were assembled with appropriate pairs of the small disks under vacuum at 900 °C for 90 min with a load of 10 MPa on a Vacuum Diffusion Bonding and Hot Pressing System. The diffusion couples were sealed into quartz capsules (evacuated and then back-flushed with argon) and then were subjected to long-term diffusion annealing at 1250 °C for 12 h followed by quenching in ice water. The diffusion couples were then sectioned in half parallel to the diffusion direction which suffered no oxidation and evaporation of elements, mounted, and polished by standard metallographic techniques. The local composition was analyzed by EPMA on JEOL JXA 8900 (JEOL, Tokyo, Japan) at the assigned area.

In order to avoid oxidation, the samples were sealed into quartz capsules again before thermal/mechanical treatment for introducing the β-to-α phase transformation process. The diffusion couples were the first solutions above α/β phase transformation temperature to obtain a fully β single-phase microstructure, then were subjected to furnace cooling to 700 °C followed by water quenching for precipitating the appropriate α phase. Considering the α/β phase transformation temperature of each alloy in the diffusion couple, the solid-solution treatment of the Ti/Ti-7.58Al-4.97Mo and Ti-5.04Al/Ti-1.52Mo diffusion couples were performed at 920 °C and 980 °C for 15 min, respectively.

Each specimen surface was carefully prepared by polishing with several diamond pastes of decreasing size followed by a final polishing with colloidal silica suspension of 0.02 μm. The microhardness was performed through the diffusion area under a loading of 980.7 mN in a HMV-2T microhardness tester made by SHIMADZU Corporation (Kyoto, Japan). The indents of the microhardness were also used as the markers, so that the in situ analysis could be achieved by testing the following microstructure and hardness in these markers areas. The microstructure and composition of the α phase at each microhardnessmarker were measuredby a Zeiss EVO MA15 (Zeiss, Oberkochen, Germany) Scanning Electron Microscope (SEM) with an Energy Disperse Spectroscopy (EDS), while the microstructure gradient in the central diffusion zone was determined by EBSD using a FEI Helios NanoLab 600i (FEI, Hillsboro, OR, USA) with an HKL-Channel 5 system. The hardness of the α phase at each microhardness marker was indented using a Hysitron TI950 triboindenter (Hysitron, Minneapolis, MN, USA) equipped with feedback control and a Cube tip. Considering the width of the α-laths was from about 500 nm to 3 μm, we used an indentation depth as small as 100 nm in every marked area. Indentations were carried out in displacement control using a trapezoidal loading curve, with a loading and unloading time of 10 s and 2 s, respectively, and a 5-s hold time at maximum depth. Then, the locations of the 100-nm-deep indents were examined by HR-SEM in FEI Helios NanoLab 600i system. EBSD was also performed on the FEI Helios NanoLab 600i system with an HKL-Channel 5 system.

## 3. Results

**Figure 1:**
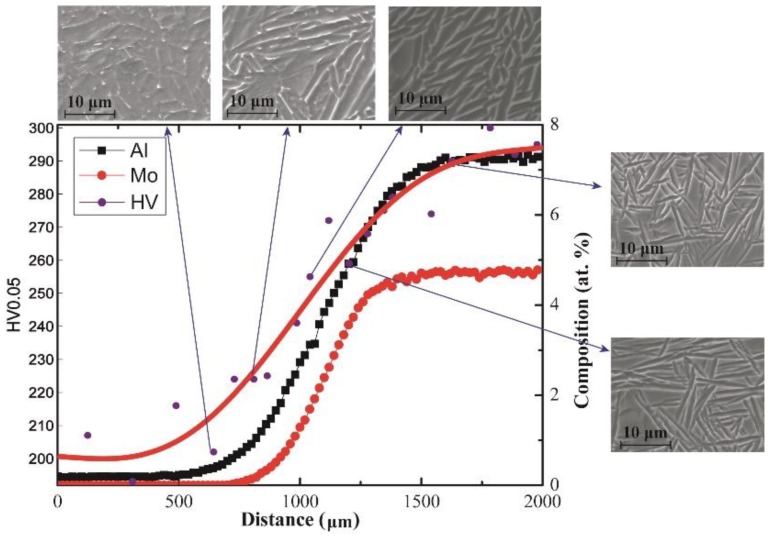
The quantitative composition–microstructure–property in Ti/Ti-7.58Al-4.97Mo diffusion couple.

**Figure 2:**
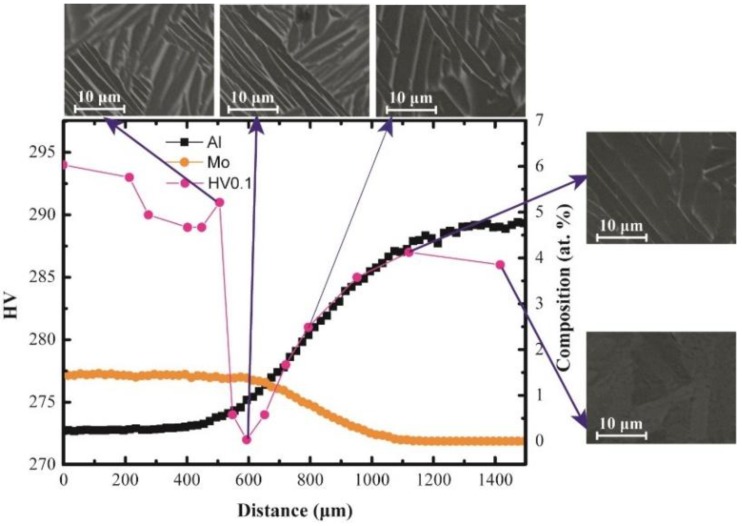
The quantitative composition–microstructure–property in Ti-5.04Al/Ti-1.52Mo diffusion couple.

After long-term diffusion annealing at 1250 °C for 12 h followed by quenching in ice water, the concentration profiles of the Ti/Ti-7.58Al-4.97Mo and Ti-5.04Al/Ti-1.52Mo diffusion couples were first measured by EPMA, as shown in Figure 1 and Figure 2. It can be seen that the long-term diffusion annealing results in a large compositions gradient of about 1000 μm in the Ti/Ti-7.58Al-4.97Mo and Ti-5.04Al/Ti-1.52Mo diffusion couples, which is suitable for distinguishing hundreds of the alloys. Based on those compositions gradients, the heat treatment is performed to the formation of the β-to-α phase transformation.

**Figure 3:**
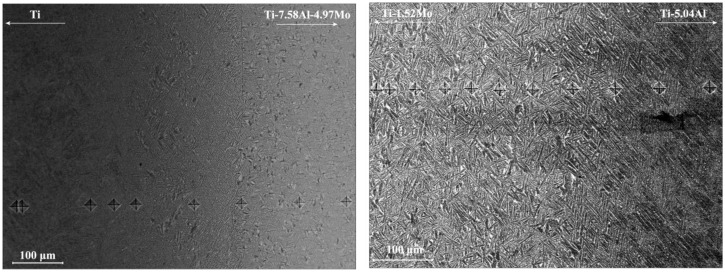
Compositional dependence of the variation of the microstructure in diffusion couples after 12 h annealing at 1250 °C followed by the heat treatment: (**a**) Ti/Ti-7.58Al-4.97Mo and (**b**) Ti-5.04Al/Ti-1.52Mo.

**Figure 4:**
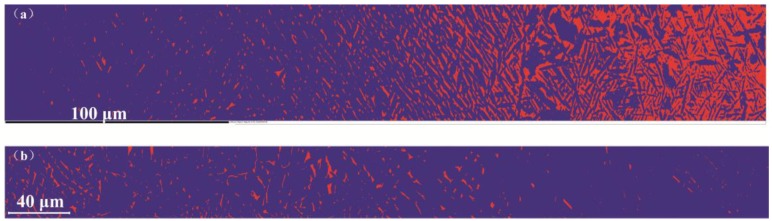
The gradient variation of the phase transformation in (**a**) Ti/Ti-7.58Al-4.97Mo and (**b**) Ti-5.04Al/Ti-1.52Mo diffusion couples. The blue represents the α phase while the red represents the β phase.

**Figure 5:**
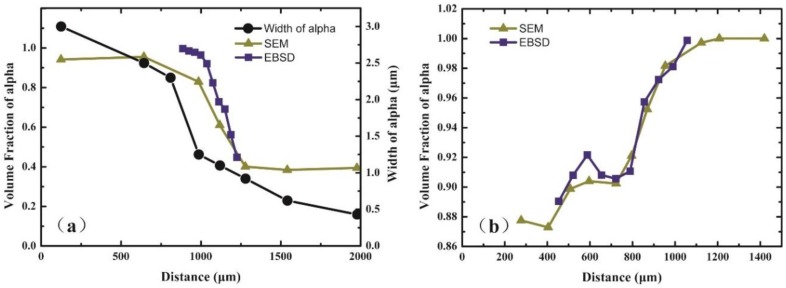
The composition-dependent microstructure characterization of the α phase in (**a**) Ti/Ti-7.58Al-4.97Mo and (**b**) Ti-5.04Al/Ti-1.52Mo diffusion couples.

Figure 3a,b shows the microstructure in the diffusion area of the Ti/Ti-7.58Al-4.97Mo and Ti-5.04Al/Ti-1.52Mo diffusion couples after heat treatment. The significant microstructure gradients have been found from one side of the diffusion couple to another side. The microstructural characterization at the Ti side of the Ti/Ti-7.58Al-4.97Mo diffusion couple reveals a full α phase, while at the Ti-7.58Al-4.97Mo side, a two-phase microstructure is observed. From the Ti side to the Ti-7.58Al-4.97Mo side, the volume fraction and the width of the α phase present gradually decrease. The EBSD results in the diffusion area of the Ti/Ti-7.58Al-4.97Mo and Ti-5.04Al/Ti-1.52Mo diffusion couples are shown in Figure 4a,b. The variation of the volume fraction from one side of the diffusion couple to another side is clearly observed. Then, by using the Image Pro Plus software, the quantitative microstructural characterization, including the volume fraction and the width of the α phase, has been measured as shown in Figure 5a,b.

By using the microhardness tester, the hardness of the matrix from one side of the diffusion couple to another side has been measured and shows in Figure 1 and Figure 2. It can be seen in Figure 1 that the hardness of the matrix increases from the Ti side to the Ti-7.58Al-4.97Mo side in the Ti/Ti-7.58Al-4.97Mo diffusion couple, while for the Ti-5.04Al/Ti-1.52Mo diffusion couple, the hardness of the matrix first decreases and then increases from the Ti-1.52Mo side to the Ti-5.04Al side.

**Figure 6:**
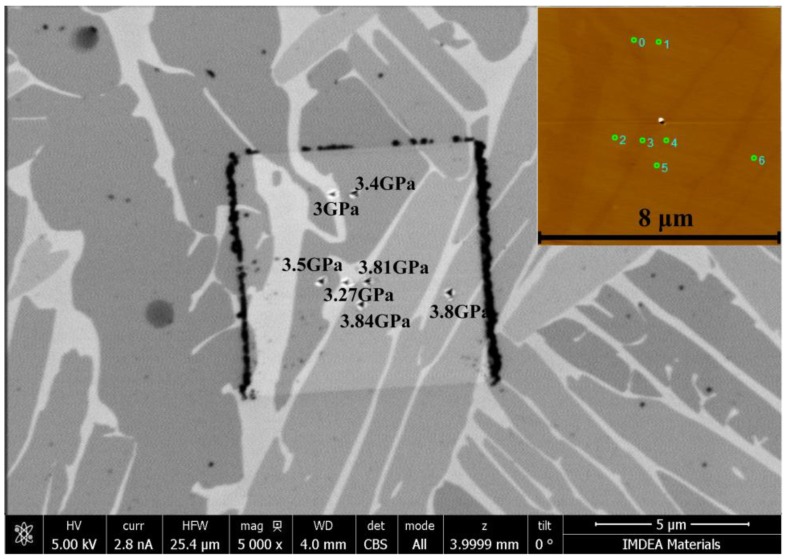
Analysis of the hardness of the α phase in Ti-5.04Al/Ti-1.52Mo diffusion couple by using the nanoindentation combined with HRSEM.

**Figure 7:**
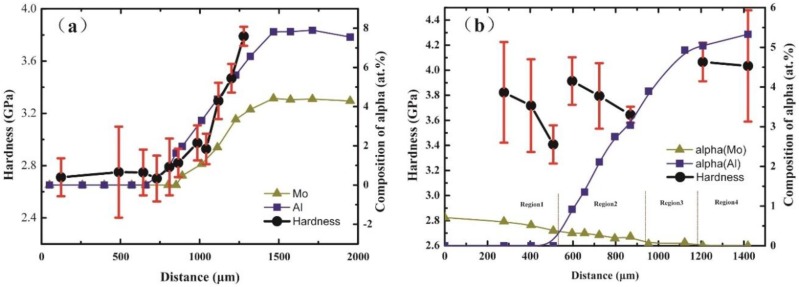
The composition-dependent hardness of the α phase in (**a**) Ti/Ti-7.58Al-4.97Mo and (**b**) Ti-5.04Al/Ti-1.52Mo diffusion couples.

An example of the nanoindentation test is shown in Figure 6. The positions of the indents are first chosen based on the Atomic Force Microscope (AFM) maps (the top left corner of Figure 6), then, after the nanoindentation test, the HR-SEM is performed to confirm the positions. It can be seen in Figure 6 that the hardness of the α phase is higher than the β phase. The average hardness of the α phase with the standard deviation in each marked position of the microhardness tester is calculated and shows in Figure 7a,b. As shown in Figure 7a, the hardness of the α phase increases from the Ti side to the Ti-7.58Al-4.97Mo side in the Ti/Ti-7.58Al-4.97Mo diffusion couple. However, a very complicated variation of the hardness of the α phase is observed in the Ti-5.04Al/Ti-1.52Mo diffusion couple from the Ti-1.52Mo side to the Ti-5.04Al side, as shown in Figure 7b.

Finally, the composition of the α phase in each marked position is measured by using the Energy Disperse Spectroscopy (EDS), as shown in Figure 7a,b. 

## 4. Discussion

### 4.1. The Compositional Dependent Microstructure and Hardness Characteristics of Alpha

The Image Pro Plus software is used for statistics of the volume fraction and the width of the α phase in each marker in Figure 3a,b, shown in Figure 5a,b. However, due to the existence of the interface of the α phase and β phase, the statistics results are later on confirmed by the EBSD analysis, shown in Figure 4a,b. It can be seen from Figure 5a that the volume fraction of the α phase decreases from 100% to 40% while the width of the α phase decreases from 3 μm to 0.5 μm from the Ti-side to Ti-7.58Al-4.97Mo-side in the Ti/Ti-7.58Al-4.97Mo diffusion couple. In the Ti-5.04Al/Ti-1.52Mo diffusion couple, the results show that the volume fraction of the α phase increases from 87% to 100% from the Ti-5.04Al-side to the Ti-1.52Mo-side, as shown in Figure 5b.

The average hardness with the standard deviation of the α phase as well as the composition variation of the α phase of the Ti/Ti-7.58Al-4.97Mo and Ti-5.04Al/Ti-1.52Mo diffusion couples are shown in Figure 7a,b. It can be seen in Figure 7a that the hardness of the α phase increases with increasing composition of Al and Mo in the α phase in the Ti/Ti-7.58Al-4.97Mo diffusion couple. Meanwhile, the complicated variation of the hardness of the α phase is observed in the Ti-5.04Al/Ti-1.52Mo diffusion couple due to the cross variation of the composition of Al and Mo in the α phase.

As shown in Figure 7b, the variation of the hardness of the α phase can be divided into four regions. In Region 1, the hardness of the α phase decreases from 3.82 GPa to 3.38 GPa when the composition of Mo in the α phase decreases from 0.71% to 0.52%. In addition, there is no Al in Region 1. However, the Al starts to appear and its composition increases from 0% to 3.04% in Region 2, while the composition of Mo in the α phase is found to continue decreasing from 0.52% to 0%. This cross variation of the composition of Al and Mo results in the decrease of the hardness of the α phase from 3.9GPa to 3.65GPa, indicating that the solution-strengthening effects of Mo is larger than Al in the α phase. It is also found that the hardness of the α phase increases from 3.38GPa to 3.9GPa when changing from Region 1 to Region 2, which should be caused by the appearance of the Al in the α phase. Combining the variation of the hardness of the α phase in Region 2, it can be concluded that the solution-strengthening of Al in the α phase demonstrates a parabolic trend, which means that the solution-strengthening effects are apparent when the composition of Al is less, while the solution-strengthening effects weaken when composition of Al in the α phase increases. Also, as there is no hardness data in Region 3, it can be speculated that the hardness of α should increase due to the increasing of the Al. As for Region 4, the composition of Al and Mo in the α phase becomes a constant value, resulting in the well-maintained hardness.

### 4.2. The Quantitative Composition–Microstructure–Property of the Alloys

The microhardness tester is used for obtaining the hardness of the matrix, shown in Figure 1 and Figure 2, together with the composition and microstructure variation of the matrix. In the Ti/Ti-7.58Al-4.97Mo diffusion couple, the increased composition of Al and Mo from the Ti side to the Ti-7.58Al-4.97Mo side will lead to the solution-strengthening effects in both α and β phases. From the Ti side to the Ti-7.58Al-4.97Mo side, the gradual decrease of the volume fraction and the width of the α phase will result in the decrease of the hardness of the matrix due to the higher hardness of the α phase in Figure 6. However, the changing of the morphology of α from lamellar to needle-like will generate the stress concentration, which will cause the enhancing of the hardness of the matrix. Those combined effects of the solution-strengthening and the microstructure change finally result in the increasing of the hardness of the matrix from the Ti side to the Ti-7.58Al-4.97Mo side.

However, the hardness of the matrix shows firstly a decrease and then an increase in the Ti-5.04Al/Ti-1.52Mo diffusion couple from the Ti-5.04Al side to the Ti-1.52Mo side. Two regions can be divided based on the microstructure change, as shown in Figure 5b. The hardness of the matrix is determined by both the factors of microstructure and solution-strengthening. The volume fraction of α in the region from 0 to 800 μm is changed only from 88% to 90% as shown in Figure 5b, which means the microstructure in this region is almost the same. Figure 2 also shows almost the same microstructure characterization in the region from 0 to 800 μm, which indicates that only the solution-strengthening will affect the hardness of the matrix in this area. It can be seen that the minimum hardness of the matrix in Figure 2 at the position of 600 μm is exactly corresponding to the minimum hardness of α in Figure 7b. In the region from 800 to 1200 μm, also the hardness of α shows a little fluctuation in Figure 7b, due to the increase of the volume fraction of α from 88% to 100%, and the hardness of the matrix increases, which indicates that the hardness of the matrix is mainly determined by the precipitation-strengthening of α. 

It can be concluded that the hardness of the titanium alloy is determined by both the solution-strengthening and precipitation-strengthening of α.

### 4.3. Accelerated Mapping of Critical Materials Data by KDC

In conclusion, many critical materials data in the Ti–Al–Mo system can be rapidly screened by the KDC method, including the quantitative composition–microstructure–property shown in Figure 1 and Figure 2, as well as the solution-strengthening effects of Al and Mo in the α phase shown in Figure 7a,b, which are necessary for accelerating the titanium alloy design.

## 5. Conclusions

In this paper, a new high throughput method named “kinetic diffusion couple” (KDC) was designed for rapidly screening many critical materials data in the Ti–Al–Mo system, including the quantitative composition–microstructure–property, as well as the solution-strengthening effects of the α phase. The results are summarized as follows:

(1) The solution-strengthening effects of Mo were found to be larger than Al in the α phase. It was also found that the solution-strengthening of Al in the α phase demonstrated a parabolic trend, which meant that the solution-strengthening effects were apparent when the composition of Al was less, while the solution-strengthening effects weakened when the composition of Al in the α phase increased.
(2) The hardness of the titanium alloy was found to be determined by both the solution-strengthening and precipitation-strengthening of α.

## References

[1] Nyakana S.L., Fanning J.C., Boyer R.R. (2005). Quick reference guide for β titanium alloys in the 00s. J. Mater. Eng. Perform..

[2] Ivasishin O.M., Markovsky P.E., Matviychuk Y.V., Semiatin S.L., Ward C.H., Fox S. (2008). A comparative study of the mechanical properties of high-strength β-titanium alloys. J. Alloys Compd..

[3] Van Bohemen S., Kamp A., Petrov R., Kestens L., Sietsma J. (2008). Nucleation and variant selection of secondary α plates in a β Ti alloy. Acta Mater..

[4] Shi R., Dixit V., Fraser H.L., Wang Y. (2014). Variant selection of grain boundary α by special prior β grain boundaries in titanium alloys. Acta Mater..

[5] Reed R.C., Tao T., Warnken N. (2009). Alloys-By-Design: Application to nickel-based single crystal superalloys. Acta Mater..

[6] Springer H., Raabe D. (2012). Rapid alloy prototyping: Compositional and thermo-mechanical high throughput bulk combinatorial design of structural materials based on the example of 30Mn-1.2C-xAl triplex steels. Acta Mater..

[7] Springer H., Belde M., Raabe D. (2013). Bulk combinatorial design of ductile martensitic stainless steels through confined martensite-to-austenite reversion. Mater. Sci. Eng. A.

[8] Zhao J.C., Jackson M.R., Peluso L.A. (2004). Mapping of the Nb-Ti-Si phase diagram using diffusion multiples. Mater. Sci. Eng. A.

[9] Chen Y., Tang B., Xu G., Wang C., Kou H., Li J., Cui Y. (2014). Diffusion research in BCC Ti-Al-Mo ternary alloys. Metall. Mater. Trans. A.

[10] Zhao J.-C. (2005). The Diffusion-Multiple Approach to Designing Alloys. Annu. Rev. Mater. Res..

[11] Zhao J.-C. (2006). Combinatorial approaches as effective tools in the study of phase diagrams and composition-structure-property relationships. Prog. Mater. Sci..

[12] Zhang X.D., Liu L.B., Zhao J.C., Wang J.L., Zheng F., Jin Z.P. (2014). High-efficiency combinatorial approach as an effective tool for accelerating metallic biomaterials research and discovery. Mater. Sci. Eng. C.

[13] Jones N., Dashwood R., Jackson M., Dye D. (2009). β Phase decomposition in Ti-5Al-5Mo-5V-3Cr. Acta Mater..

[14] Kou H., Chen Y., Tang B., Cui Y., Sun F., Li J., Xue X. (2014). An experimental study on the mechanism of texture evolution during hot-rolling process in a β titanium alloy. J. Alloys Compd..

[15] Mao S., Wang C., Li N., Wang J., Chen Y., Xu G., Guo Y., Cui Y. (2018). Kinetic diffusion multiple: A high throughput approach to screening the composition-microstructure-micromechanical properties relationships. Calphad.

[16] Wang C., Yang L., Cui Y., Pérez-Pradoa M.T. (2017). High throughput analysis of solute effects on the mechanical behavior and slip activity of beta titanium alloys. Mater. Des..

[17] Zhao J.C., Jackson M.R., Peluso L.A., Brewer L.N. (2002). A diffusion-multiple approach for mapping phase diagrams, hardness, and elastic modulus. JOM.

[18] Zhao J.C., Zheng X., Cahill D.G. (2005). High-throughput diffusion multiples. Mater. Today.

[19] Sun Z., Guo S., Yang H. (2013). Nucleation and growth mechanism of α-lamellae of Ti alloy TA15 cooling from an α+β phase field. Acta Mater..

[20] Harper M.L. (2004). A Study of the Microstructural and Phase Evolutions in TIMETAL 555.

[21] Kattner U.R., Lin J.C., Chang Y.A. (1992). Thermodynamic assessment and calculation of the Ti-Al system. MTA.

[22] Murray J.L. (1981). The Mo-Ti (Molybdenum-Titanium) system. Bull. Alloy Phase Diagr..

[23] Cupid D.M., Fabrichnaya O., Ebrahimi F., Seifert H.J. (2010). Thermodynamic assessment of the Al–Mo system and of the Ti-Al-Mo System from 0 to 20at.% Ti. Intermetallics.

